# *Momordica charantia* polysaccharides modulate the differentiation of neural stem cells via SIRT1/Β-catenin axis in cerebral ischemia/reperfusion

**DOI:** 10.1186/s13287-020-02000-2

**Published:** 2020-11-16

**Authors:** Zhaoli Hu, Fengying Li, Xiaoling Zhou, Feng Zhang, Linyan Huang, Bing Gu, Jiangang Shen, Suhua Qi

**Affiliations:** 1 Research Center for Biochemistry and Molecular Biology and Jiangsu Key Laboratory of Brain Disease Bioinformation, Xuzhou Medical University, Xuzhou, China; 2 School of Medical Technology, Xuzhou Medical University, Xuzhou, China; 3 Department of Laboratory Medicine, Affiliated Hospital of Xuzhou Medical University, Xuzhou, China; 4 School of Chinese Medicine, The University of Hong Kong, Hong Kong, China

**Keywords:** *Momordica charantia* polysaccharides (MCPs), Neural stem cells (NSCs), Differentiation, SIRT1, β-Catenin

## Abstract

**Background:**

Stroke is the leading cause of long-term motor disability and cognitive impairment. Recently, neurogenesis has become an attractive strategy for the chronic recovery of stroke. It is important to understand the molecular mechanism that promotes neural stem cell (NSC) neurogenesis for future NSC-based therapies. Our previous study showed that *Momordica charantia* polysaccharides (MCPs) exerted neuroprotective effects on stroke via their anti-oxidant and anti-inflammation activities. However, it remains unknown whether MCPs promote NSC neurogenesis after cerebral ischemic/reperfusion injury (IRI).

**Methods:**

We investigated MCPs’ function in differentiation of neural stem cells (NSCs) in vivo and in vitro experiments. Based on a middle cerebral artery occlusion (MCAO) rat model, the effect of MCPs on neuronal differentiation after MCAO was analyzed. Primary NSCs and neural stem cell line C17.2 were cultured and subjected to glutamate stimulation to establish the cell model of IRI. We evaluated the effect of MCPs on NSC differentiation in IRI cell model by Western blot and immunofluorescence staining. The SIRT1 activity of NSCs post glutamate stimulation was also evaluated by CELL SIRT1 COLORIMETRY ASSAY KIT. In addition, molecular mechanism was clarified by employing the activator and inhibitor of SIRT1.

**Results:**

MCPs had no effects on the differentiation of neural stem cells under physiological conditions while shifted NSC differentiation potential from the gliogenic to neurogenic lineage under pathological conditions. Activation of SIRT1 with MCPs was responsible for the neuronal differentiation of C17.2-NSCs. The neuronal differentiation effect of MCPs was attributed to upregulation SIRT1-mediated deacetylation of β-catenin. MCP-induced deacetylation via SIRT1 promoted nuclear accumulation of β-catenin in NSCs.

**Conclusion:**

Our findings indicate that the deacetylation of β-catenin by SIRT1 represents a critical mechanism of action of MCPs in promoting NSC neuronal differentiation. It provides an improved understanding of molecular mechanism underlying neuroprotective effects of MCPs in IRI, indicating its potential role on treating ischemic stroke especially chronic recovery.

## Introduction

Stroke is a major disease that affects millions of people annually across the world [1–3]. As far as we have known that, after the onset of cerebral ischemia, a set of time-dependent cellular events are activated including not only glutamate receptor-mediated excitotoxicity, apoptosis, and blood-brain barrier (BBB) dysfunction and inflammation but also endogenous neural repair [4, 5].

Accumulating evidence has suggested that the neurogenesis occurs in rodents, as well as in primate and patients after ischemic stroke [6–8]. However, the spontaneous repair capacity after cerebral ischemia is limited because proximately 80% of the new neurons produced by ischemia die within 2 weeks, and only a very few populations can survive over a long period of time [9, 10]. Therefore, pharmacological interventions related to promoting endogenous neurogenesis and prolonging the survival of differentiated neurons after stroke are believed to be a promising strategy for brain repair.

*Momordica charantia* (MC), belonging to the gourd family, is an important multipurpose edible, medicinal plant widely distributed throughout Asia. In recent study, we have provided experimental evidence for that polysaccharide is main effective components extracted from MC. Additionally, *Momordica charantia* polysaccharides (MCPs) have a protective role on nerve injury after stroke by scavenging free radicals [11]. The other teams’ previous research also demonstrated that MCPs have high anti-oxidant capacity for scavenging reactive oxygen species (ROS) [12, 13]. The side effects of currently prescribed synthetic anti-oxidants have prompted the use of alternative traditional herbal medicines and dietary supplements anti-oxidants associated with fewer adverse effects in the treatment of ischemia stroke [14]. It is well established that variety of natural molecules present in the diet, such as plant polysaccharide, could protect the brain and delay aging. Emerging studies show a role for anti-oxidant-rich foods such as fruits, vegetables, and nuts in improving cognitive damage by preventing or delaying the onset of cognitive decline during aging and neurodegeneration [15, 16]. As naturally compounds found in daily foods, MCPs are well known for their anti-oxidation, anti-inflammation, anti-tumor, hypoglycemic, and anti-diabetic effects [17]; however, little is known about its effects from neurogenesis regulation perspective.

Silent information regulator factor 2-related enzyme 1 (sirtuin1, SIRT1) is a deacetylase which is widely expressed in the whole adult brain [18] and has been shown to interact with set of protein targets involved in Wnt signaling [19], glucose homeostasis [20, 21], and calcium signaling [22, 23], making SIRT1 an attractive candidate target for controlling senescence, feeding behavior, energy expenditure, and oxidative stress [24]. By deacetylating a variety of proteins, SIRT1 can mediate a broad range of vital biological functions including gene expression, DNA repair and apoptosis, neurogenesis, and aging. Thus, SIRT1 promotes cellular longevity through a number of mechanisms [25]. Studies of the aging brain have shown that the expression of SIRT1 decline with age, which is accompanied by a higher incidence of aging related diseases in mammals, such as ischemic stroke [26–28]. In addition, SIRT1 has been found to delay aging and considered to be one of the determining factors of the biology of stem cells, including neural stem cells (NSCs) and neural progenitor cells (NPCs). SIRT1 has been implicated in NSCs proliferation and differentiation through deacetylate variety of histone and non-histone proteins and transcription factors [29, 30]. These phenomena raise the possibility that SIRT1 may play a crucial role on neurogenesis after ischemic stroke.

During recent years, canonical Wnt/β-catenin signaling has been found to play a key role in controlling the maintenance of neural stem cells and the development of neural tissues and progenitor cells [31]. The accumulation of β-catenin in the nucleus is the key step in canonical Wnt/β-catenin signaling pathway [32]. Furthermore, the post-translational modification of β-catenin, including phosphorylation and deacetylation, is an important mechanism of regulating of β-catenin stability and subcellular location, as well as its transcriptional activity [33, 34]. However, it has not previously been explored whether MCPs regulate nuclear translocation of β-Catenin via SIRT1 to promote differentiation of NSCs after stroke. Therefore, in the present study, we aim to determine whether MCPs could improve NSC differentiation and to further explore whether SIRT1/β-catenin axis plays roles in post-stroke neurogenesis.

## Material and methods

### MCPs preparation

MCPs (purity ≥ 99%) were extracted from *M. charantia* by water extraction and alcohol precipitation, followed by the elimination of proteins and starch. Studies in our team [11] have identified MCPs composition by high performance liquid chromatography (HPLC) and mass spectrometry (MS). HPLC-MS/MS analysis revealed that galacturonic acid was the major ingredients of MCP accounted for 93.7% and rhamnose, xylose, mannose, glucose, and galactose were in the molar ratio of 0.06%, 0.18%, 0.03%, 0.8%, and 0.28%, respectively. The extraction method was provided by Professor Min Wang from China Pharmaceutical University.

### Antibody and reagents

The following primary antibodies were used in this study: mouse anti-SIRT1 antibody [19A7AB4] (1 μg/ml, ab110304, Abcam), rabbit anti-β-catenin antibody [E247] (1:5000; ab32572, Abcam), rabbit anti-BrdU antibody (1:1000, B35138, Life technologies), mouse anti-beta III Tubulin antibody [TUJ-1] (1:1000, ab14545, Abcam), mouse anti-MAP 2 antibody (1:1000; ab5392, Abcam), mouse MAb anti-GFAP antibody (1:1000; ab106509, Abcam), mouse anti-CNPase antibody [11-5B] (5 μg/ml, ab6319, Abcam), mouse anti-acetyl Lysine antibody [1C6] (1:1000; ab22550, Abcam), rabbit monoclonal anti-Lamin B1 antibody (1:3000, #13435, Cell Signaling Technology, USA), and β-actin (1:5000, #4970, Cell Signaling Technology, USA). The secondary antibodies used in our experiment were goat anti-mouse IgG (1:10000) and goat anti-rabbit IgG (1:10000), which were purchased from Sigma-Aldrich (St. Louis, MO, USA). SIRT1 small interfering RNA (siRNA) (#5239398) was obtained from Life Technologies (USA). Resveratrol was obtained from Sigma-Aldrich (St. Louis, MO, USA). Nicotinamide was acquired from Beyotime biotechnologies (USA). Normal goat serum stock solution was purchased from ZSGB-BIO (#ZLI-9021, China).

### Middle cerebral artery occlusion (MCAO) model

Adult male Sprague-Dawley rats (240~280 g) were obtained from Shanghai Experimental Animal Center of the Chinese Academy of Science. All the surgical procedures were in accordance with the institutional guidelines, and the experimental procedures were approved by the Animal Ethics Committee of Xuzhou Medical University (approval ID, SCXK (SU) 2010-0003). The rats were maintained in standard cages in a controlled environment (temperature, 24 ± 1 °C; relative humidity, 50~60%; light period, 06:00~18:00) and given access to food and water ad libitum. The rats were used in the study after 3 days of acclimatization. The middle cerebral artery occlusion (MCAO) stroke model was performed with 5 h of MCAO plus reperfusion 14 days. Briefly, rats were anesthetized with 4% isofluorane and maintain1ed at 2% isofluorane via inhalation. In MCAO group, a silicon-coated suture with a tip diameter of 0.38 mm (Doccol, Redlands, CA, USA) was inserted from the external carotid artery (ECA) into the internal carotid artery (ICA), until its tip occluded the origin of the left middle cerebral artery (MCA). In the sham group, rats underwent the same anesthesia and surgical procedure without MCA occlusion.

### Experimental groups and drug administration

**Fig. 1:**
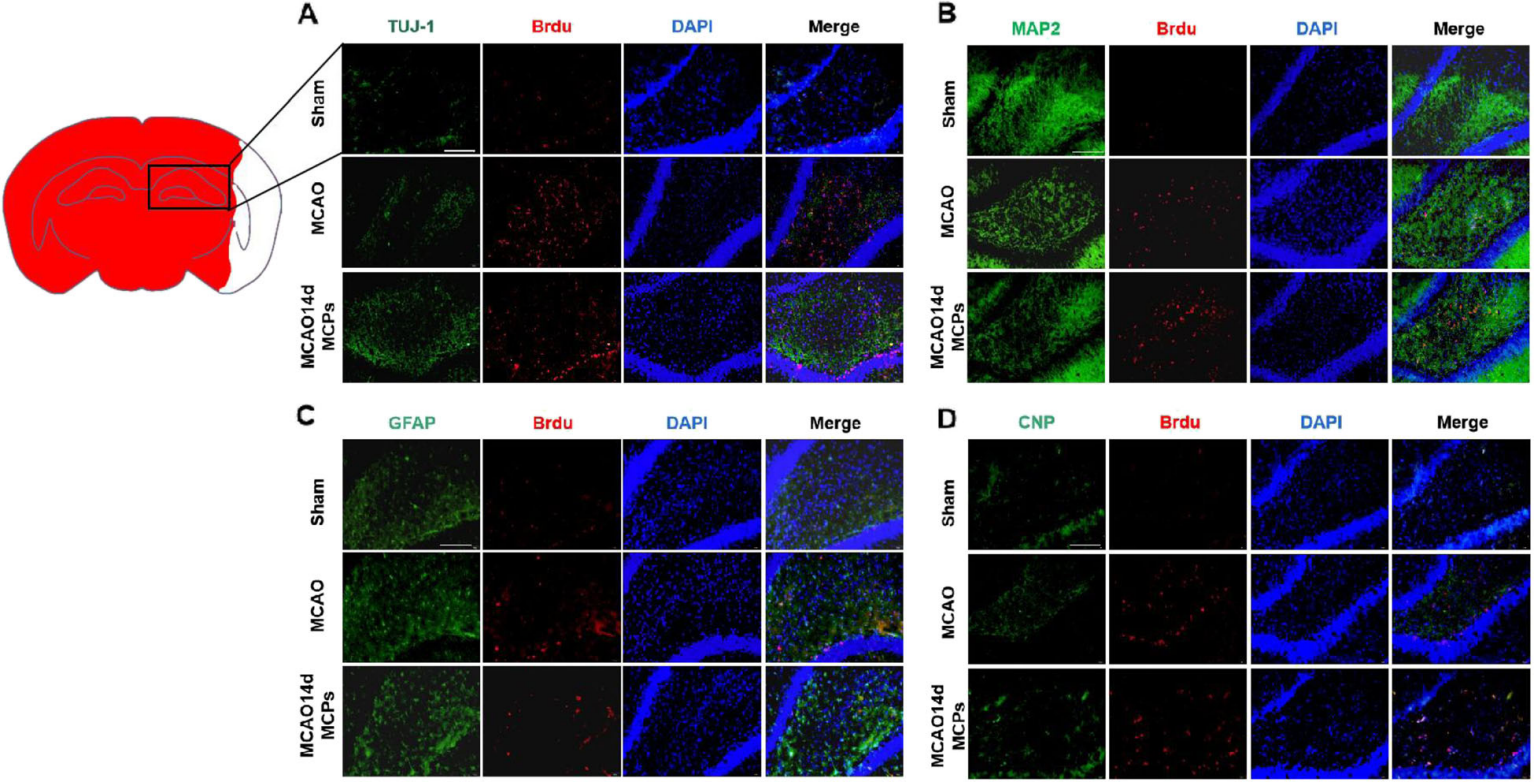
MCPs enhance neuronal differentiation of the subgranular zone (SGZ) of the hippocampus after MCAO in rats. **a** Immunofluorescence images of BrdU (red), TUJ1 (green), and DAPI (blue). **b** Immunofluorescence images of BrdU (red), MAP 2 (green), and DAPI (blue). **c** Immunofluorescence images of BrdU (red), GFAP (green), and DAPI (blue). **d** Immunofluorescence images of BrdU (red), CNP (green), and DAPI (blue) in SGZ region of MACO rats treated with MCPs (*n* = 5 rats) from indicated group; scale bar = 200 μm

All treatments were administered in a blinded manner. Rats were randomly divided into 3 groups with six mice per group (Fig. 1): (1) sham operation rats, (2) MCAO rats, and (3) MCAO rats treated with MCPs (200 mg/kg) after MCAO. The rats treated with MCPs were subjected to intragastric administration of MCPs (200 mg/kg) every 24 h for 2 weeks after MCAO.

### Brain section immunofluorescence labeling

Anesthetized rats were perfused through the left ventricle with ice saline followed by ice 4% paraformaldehyde in 0.1 M sodium phosphate buffer (pH 7.4). Brains were quickly removed and fixed with the same fixation solution overnight at 4 °C. The tissue was gradient dehydrationed with 20% and 30% sucrose for 24–48 h. Twenty-micrometer coronal sections were taken from fresh frozen mouse brains. After washing with 0.01 M PBS, the slices were incubated with blocking buffer (PBS with 10% goat serum and 0.5% Triton X-100) for 2 h at RT. For in situ hybridization, double immunofluorescence was used to co-localize BrdU plus Tuj1 (MAP 2, GFAP, or CNP) in the same slices overnight at 4 °C. Then, appropriate fluorescent antibody was added and incubated at room temperature for 1 h followed by staining of the nuclei with DAPI (10 mg/mL). Images were obtained with a digital microscope (Olympus CKX53, Japan) and analyzed by Image-Pro Plus 6.0 software.

### Cell culture

C17.2 cells are an immortalized NSC line originally derived from external germinal layer of mouse cerebellum. For the unique ability to self-renew and generate both neurons and glia in vitro, this cell line is predominately used as a model for multipotent stem cells [35]. In the present study, C17.2 cells were kindly provided by Professor Jiangang Shen (School of Chinese Medicine, Li Ka Shing Faculty of Medicine, The University of Hong Kong, 10 Sassoon Road, Pokfulam, Hong Kong, SAR, China). C17.2 cells were maintained in Dulbecco’s modified Eagle’s medium (DMEM; Invitrogen) containing 10% fetal bovine serum (FBS, Gibco) and 2 mM L-glutamine at 37 °C in humidified incubators with a 5% CO_2_ atmosphere and passaged at 50% confluence every 2 days.

### Establishment of ischemia reperfusion cell model and drug treatment

Glutamate is the major excitatory neurotransmitter in the central nervous system. Early events following ischemic damage include reactive oxygen species (ROS)-mediated oxidative stress and glutamate-induced excitotoxicity, both of which contribute to rapid cell death within the infarct core. Exposure to glutamate, implicated in damage induced by oxidative stress, is usually used as an in vitro model of ischemia/reperfusion-induced cell death. For mimic this damage in vitro, the model of injured NSCs by glutamate was built up. The detailed method was described as previously reported [36, 37]. Briefly, for C17.2 cells, glutamate-mediated neurotoxicity was induced by 30 min exposure to 100 μM glutamate in the normal feeding medium. For restoration, cultured C17.2 cells were rinsed twice with PBS and the original feeding medium was restored for different period of times (0 days, 1 day, 3 days, 4 days, 5 days, 14 days) to establish glutamate-induced insults in vitro.

For drug treatments, MCPs at various concentrations (1.0, 2.0, 3.0, 5.0, 10.0, and 20.0 μg/ml) were added into cell cultures for 24 h after the end of glutamate exposure. To activate or inhibit Sirt1, C17.2 cells were pre-incubated with resveratrol (Sirt1 activators, 25 μM) or nicotinamide (Sirt1 inhibitors, 25 μM) for 24 h prior to glutamate exposure.

### Primary cortical neural stem cells (E16-NSC) culture

All procedures involving the use of animals were approved by the local ethical review committee. Primary cortical neural stem cells (E16-NSC) were isolated from cerebral cortexes of Sprague-Dawley rat embryo on E16. Briefly, whole cerebral neocortices were removed from the rat fetuses, then mechanically dissociated. The single-cell suspension was transfected to 6-cm culture dishes and cultured as floating neurospheres in a humidified atmosphere with 5% CO_2_ at 37 °C. Neurospheres were maintained in neural-basal medium (DMEM/F-12, 1:1, Hyclone), which was supplemented with B27-supplement, 20 μg/l basic bFGF and 20 ng/ml EGF (both from Invitrogen), 2 mmol/L-glutamine (Invitrogen), and 10,000 U/l penicillin and 10 mg/l streptomycin (both from Hyclone). After 5–7 days, the neurospheres were trypsinised (0.05% trypsin with 0.02% EDTA, Sigma-Aldrich) as single-cell and seeded in poly-D-lysine-treated culture plates at a density of 5 × 10^4^ cells. Then, cells were cultured for 2 to 3 days at 37 °C in a 7.5% CO_2_ atmosphere and used for in vitro experiment.

### siRNA transfection

Small interfering RNAs (siRNAs, 5239398) targeting sitr1 were obtained from Life Technologies. Six-well plates were plated with C17.2-NSCs for transfection, and when cells reached 60 to 70% confluence, the lipofectamine 3000 (Invitrogen, Carlsbad, CA, USA) were used to transfected cells with either SIRT1-specific small interfering RNA (siRNA) (50 nM) or negative control siRNA (50 nM) mixed with Lipofectamine RNAiMAX (all from Life Technologies) according to the manufacturer’s instructions. All transfected cells were harvested at 48 h after transfection for subsequent experiment.

### Cellular immunofluorescence staining

For differentiation detection, the expression of Tuj1, GFAP, and CNP were analyzed by cellular immunofluorescence staining. The cells were seeded on glass coverslips in DMEM supplemented with 10% FBS. Coverslips were rinsed with PBS and then fixed with 4% paraformaldehyde solution for 10 min at room temperature (RT). After fixation, the cells were incubated in blocking buffer (1× PBS with 5% normal goat serum stock solution) for 1 h at RT. Then, cells were rinsed three times with 0.01 M PBS (pH 7.4), incubated with the primary antibody (anti-Tuj1, anti-GFAP, or anti-CNP), overnight at 4 °C, washed with PBS for three times, and then incubated with the secondary antibody (Alexa Fluor 488-conjugated goat anti-mouse antibody, 1:200, Invitrogen) for 2 h at RT, and DAPI (10 mg/mL) was added 15 min to display the nuclei. Images of 10 randomly selected fields of view were captured under a fluorescence microscope.

### Protein sample and subcellular protein sample preparation

The cells were washed three times with iced PBS and lysed using 300~400 μL lysis buffer (containing 50 mM Tris-HCl, 140 mM NaCl, 1.5 mM MgCl_2_, 0.5% NP-40, 1 mM Na_3_VO_4_, 1 mM p-nitrophenyl phosphate, 0.5 mM PMSF, 10 μg/mL leupeptin, 10 μg/mL aprotinin, and 10 μg/mL pepstatin) on ice for 30 min before collection. The mixture was then centrifuged to isolate the supernatant, which contained the cytoplasmic protein. The pellet was washed by adding 400 μL of lysis buffer and then centrifuged to discard the supernatant. The pellet was re-suspended with 100 μL of RIPA lysate (containing 50 mM Tris-HCl, 150 mM NaCl, 1% Triton X-100, 1% sodium deoxycholate, 0.1% SDS, 1 mM EDTA, 1 mM Na_3_VO_4_, 1 mM p-nitrophenyl phosphate, 0.5 Mm PMSF, 10 μg/mL leupeptin, 10 μg/mL aprotinin, and 10 μg/mL pepstatin). After three ultrasonic treatments for 5 s at intervals of 5 s and centrifugation at 12,000×*g* for 20 min at 4 °C, the supernatant was the nuclear protein.

### Immunoprecipitation

Coimmunoprecipitation (co-IP) was performed according to the following procedures. Briefly, protein samples were precleared for 1 h at 4 °C using 20 μl of protein A-Sepharose CL-4B (Amersham Biosciences, Uppsala, Sweden) to remove nonspecific proteins. After centrifugation, supernatants were incubated with 1 μg of primary antibodies overnight at 4 °C. Targeted immune complexes were captured with 2 h incubation of protein A. Samples were eluted three times with immunoprecipitation buffer. Targeted proteins were eluted by boiling at 100 °C for 5 min in SDS-PAGE loading buffer and then isolated by centrifugation. Then, immunoprecipitates were subjected to Western blot analysis.

### Immunoblotting

Western blot analysis was performed according to the standard protocol. Briefly, proteins were separated by 4–12% SDS-PAGE and transferred to PVDF membranes (Merck KGaA, Darmstadt, Germany) for 90 min at 15 V. The membranes were then blocked with 5% non-fat dry milk in Tris-buffered saline and Tween 20 (TBST) and then incubated with appropriate primary antibodies at 4 °C overnight, followed by washing with TBST, then incubating with horseradish peroxidase-conjugated secondary antibodies in TBST for 1 h at RT. Then, the bands were visualized by ECL (Pierce) and detected by Bio-Rad digital imaging system (Bio-Rad Laboratories, Inc.). For quantification, the density of the bands on the membrane was quantified using Image J 1.48 software.

### Assessment of SIRT1 activity

Sirt1 deacetylase activity was determined with the CELL SIRT1 COLORIMETRY ASSAY KIT (GenMed Scientifics Inc., USA) based on FluordeLys-SIRT1 substrate peptide. Protein extracts from C17.2 cells were incubated with the fluorogenic acetylated peptide substrate. The reaction was carried out at 37 °C for 1 h, and the fluorescent signal was measured at 360 nm excitation and 460 nm emission on a fluorescence plate reader. Results were expressed as percentage of the control.

### Data analysis and statistics

All values are presented as the means ± SD. Statistical analysis of the results was carried out by one-way or two-way ANOVA, followed by Duncan’s new multiple range method or the Newman-Keuls test. *P* values < 0.05 were considered be statistically significant. All of the experiments were repeated at least three times in an independent manner.

## Results

### MCPs enhance neuronal differentiation of the subgranular zone (SGZ) after MCAO in rats

To further explore the effect of MCPs on neuronal differentiation in vivo, we performed double-labeling at day 14 after MCAO with antibodies against BrdU (red), a marker for DNA replication in newly formed cells, plus TUJ1 (green), BrdU (red) plus MAP 2 (green), BrdU (red) plus GFAP (green), or BrdU (red) plus CNP (green) to detect neurons, astrocytes, or oligodenrocytes, respectively. Analysis of the microphotographs of brain sections obtained at days 14 after surgery showed (Fig. 1a, b) that IR injury led to an increase of co-localization between BrdU (red) plus TUJ1 (green) or BrdU (red) plus MAP 2 (green) in the cellular layer. After 14-day MCP treatment, it is noted that neuronal specific marker co-localization with BrdU exhibited enhancement signal, but another two gliogenic specific marker, GFAP (green) or CNP (green), co-localization with BrdU in SGZ in the MCPs-treated animals did not differ from sham operated (Fig. 1c, d). These results suggested that MCP treatment promoted hippocampal SGZ neuronal differentiation of NSCs in MCAO rats.

### MCPs have no effects on the differentiation of neural stem cells under physiological conditions

**Fig. 2:**
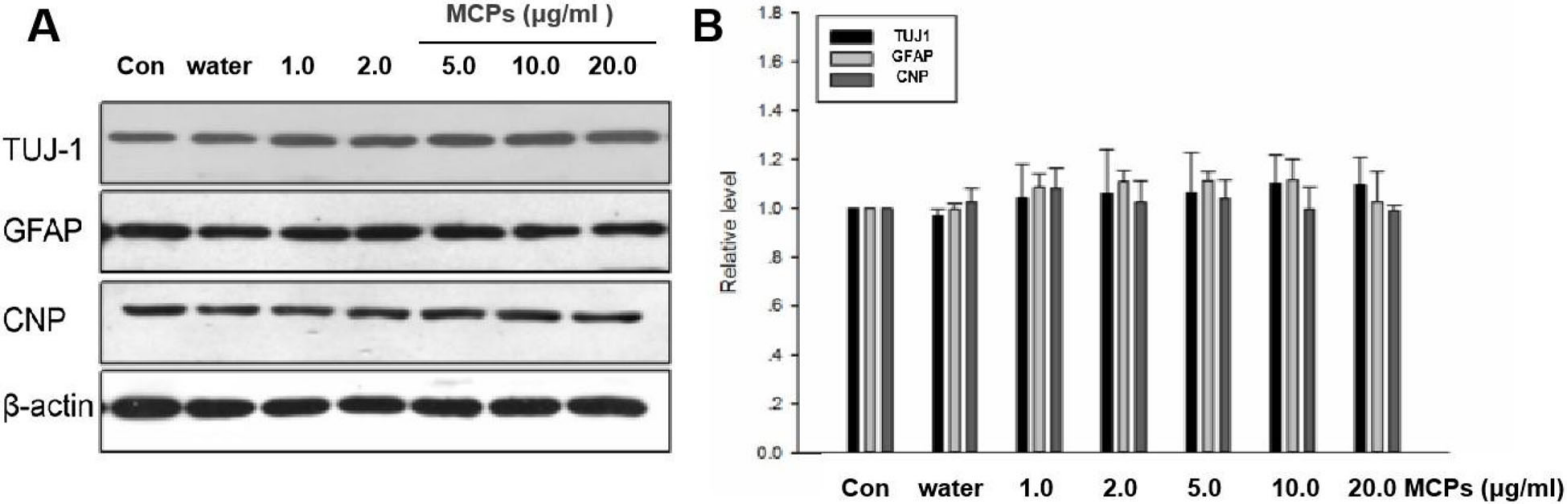
MCPs have no effects on the differentiation of C17.2-NSCs under physiological conditions. **a** Western blots for TUJ1, GFAP, and CNP from indicated group. β-actin was used for signal normalization. **b** Data are presented as the relative density of TUJ1, GFAP, and CNP compared with that of β-actin. Data were given as mean ± SD according to ANOVA with *n* = 3 independent experiments in triplicate per group

To determine whether MCP treatment affects NSC differentiation under physiological conditions, we observed the expression of three NSC differentiation marker genes, including TUJ1, GFAP, and CNP at 24 h after reperfusion in C17.2-NSCs. MCP treatment affects NSC differentiation under physiological conditions, as displayed in Fig. 2, no significant difference was discovered between the Con group and the different concentrations of MCP treatment groups (1.0, 3.0, 5.0, 10.0, and 20.0 μg/ml) in Tubulin, GFAP, or CNP expression. The data suggest that MCP treatment could not affect NSC differentiation under physiological conditions.

### MCPs shift NSC differentiation potential from the gliogenic to neurogenic lineage under pathological conditions

**Fig. 3:**
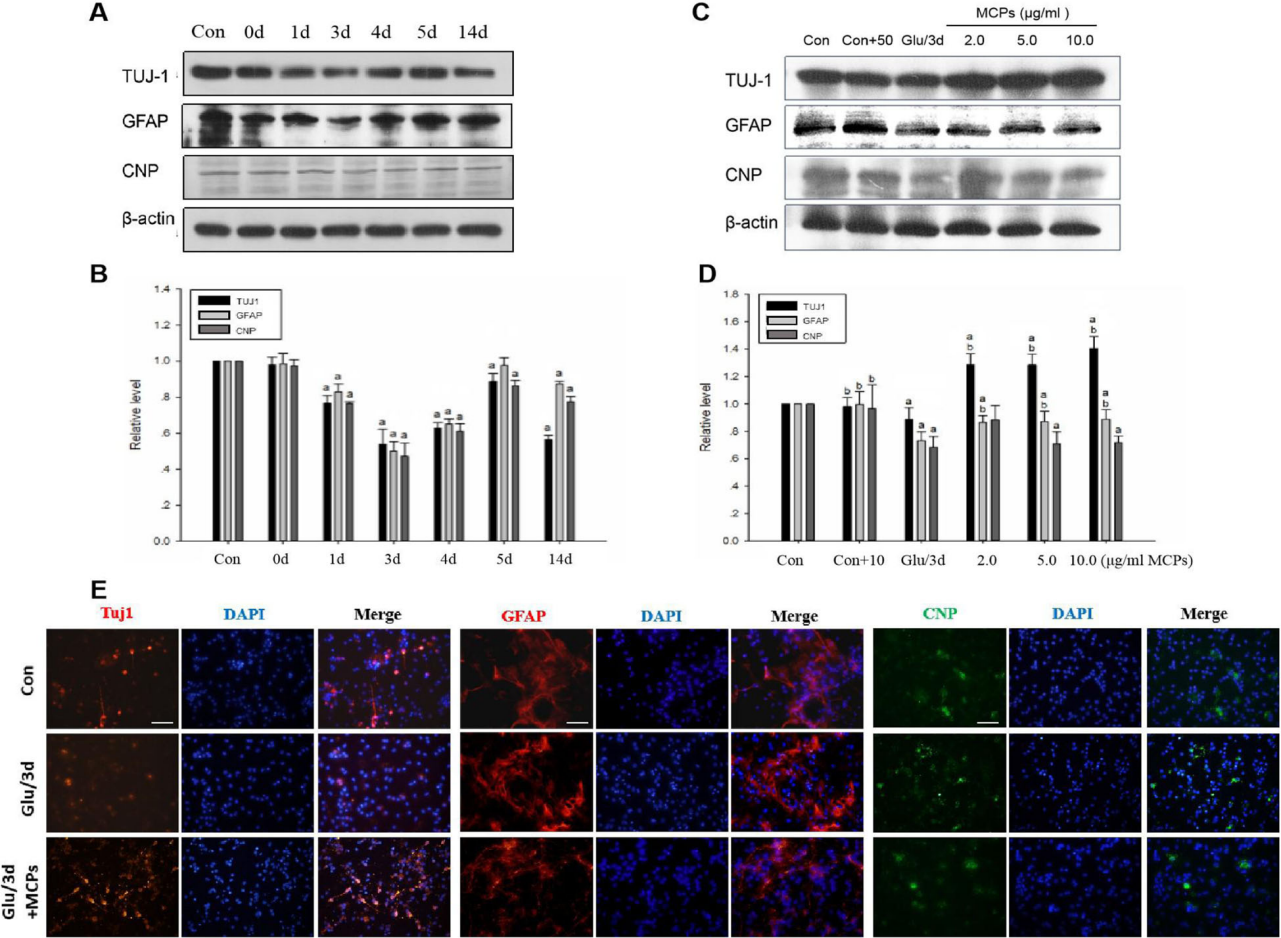
MCPs can promote the neuronal differentiation of C17.2-NSCs under pathological conditions. **a** C17.2-NSCs were treated with glutamic acid and then perfused of different periods (0 days, 1 day, 3 days, 5 days, 7 days, 14 days). Western blots for TUJ1, GFAP, and CNP from indicated group. **b** Data were presented as the relative density of TUJ1, GFAP, and CNP compared with that of β-actin. **c** C17.2-NSCs were treated with different concentration of MCP (2.0, 5.0, 10.0 μg/ml) after glutamic acid stimulation reperfusion of 3 days (Glu/3d+MCP group). Western blots for TUJ1, GFAP, and CNP from indicated group. **d** Data were presented as the relative density of TUJ1, GFAP, and CNP compared with that of β-actin. **e** Immunofluorescent staining of TUJ1 (red), GFAP (red), and CNP (green) in the indicated group. DAPI was used to stain the cell nucleus (blue). Scale bar, 100 μm. Data were given as mean ± SD according to ANOVA with *n* = 3 independent experiments in triplicate per group. ^a^*P* < 0.05 vs. control, ^b^*P* < 0.05 vs. Glu/3d group

To exclude the effect of C17.2-NSCs themselves differentiation on the experiment and to determine the time point of MCP administration, C17.2 cells were treated with 30 min of glutamate (100 μM) and then were subsequently restored for different period of times to mimic ischemic induced in vitro. Firstly, we examine differentiation of C17.2-NSCs without the presence of MCPs by monitoring the protein level of three NSC differentiation markers. The results from Western blot assays indicated that the expression of TUJ1, GFAP, and CNP gradually declined on day 0 and reached its lowest point on day 3 and then started to increase on day 5 after glutamic acid stimulation compared with the control cells (Fig. 3a, b). Upon glutamic acid treatment for 14 days, expression of TUJ1 dramatically decreased again and the other two cell markers GFAP and CNP still maintained higher level (Fig. 3a, b).

Next, as shown in Fig. 3c and d, upon MCP treatment, the expression of TUJ1 was significantly increased in different concentrations MCP treatment groups compared with that in the control group and Glu/3d group. While the expression of GFAP dramatically decreased compared with that in the control group and Glu/3d group, in contrast, no change of the protein level of CNP were found compared with the Glu/3d group (Fig. 3c, d). Taken together, these results suggested that MCPs could obviously promote C17.2-NSC differentiation into neuron under the ischemic pathological state.

To further demonstrate the effect of MCPs on NSC differentiation, primary cortical neural stem cells (E16-NSC) were isolated and identified. Immunofluorescent staining (Supplementary figure S1.) showed that cultured E16-NSCs cells were mostly stem cells, with almost no neurons, astrocytes, or oligodendrocytes, and exhibited strong proliferative ability. In addition, we also detected the differentiation profile of E16-NSC in response to glutamate-inducing stimuli (Supplementary figure S2.). The results indicated that glutamate treatment in vitro could successfully induce the change of phenotype in cultured E16-NSC. Therefore, this cell model was used for subsequent differentiation experiments. We detected three lineage-specific differentiation markers (Tuj1, GFAP, and CNP) by immunofluorescent staining (Fig. 3e). As observed in the earlier experiments shown in Fig. 3c and d, glutamate treatment reduced both Tuj1-, GFAP-, and CNP-positive cells. Upon treatment with MCPs, the number of Tuj1-positive cells was significant increased compared with glutamate treatment group. However, MCP treatment had no significant impact on the number of GFAP- or CNP-positive cells (Fig. 3e). Taken together, these findings indicated that MCPs may enhance the neuronal differentiation of E16-NSCs under oxidative stress induced by glutamate.

### Activation of SIRT1 with MCPs is responsible for the neuronal differentiation of C17.2-NSCs

**Fig. 4:**
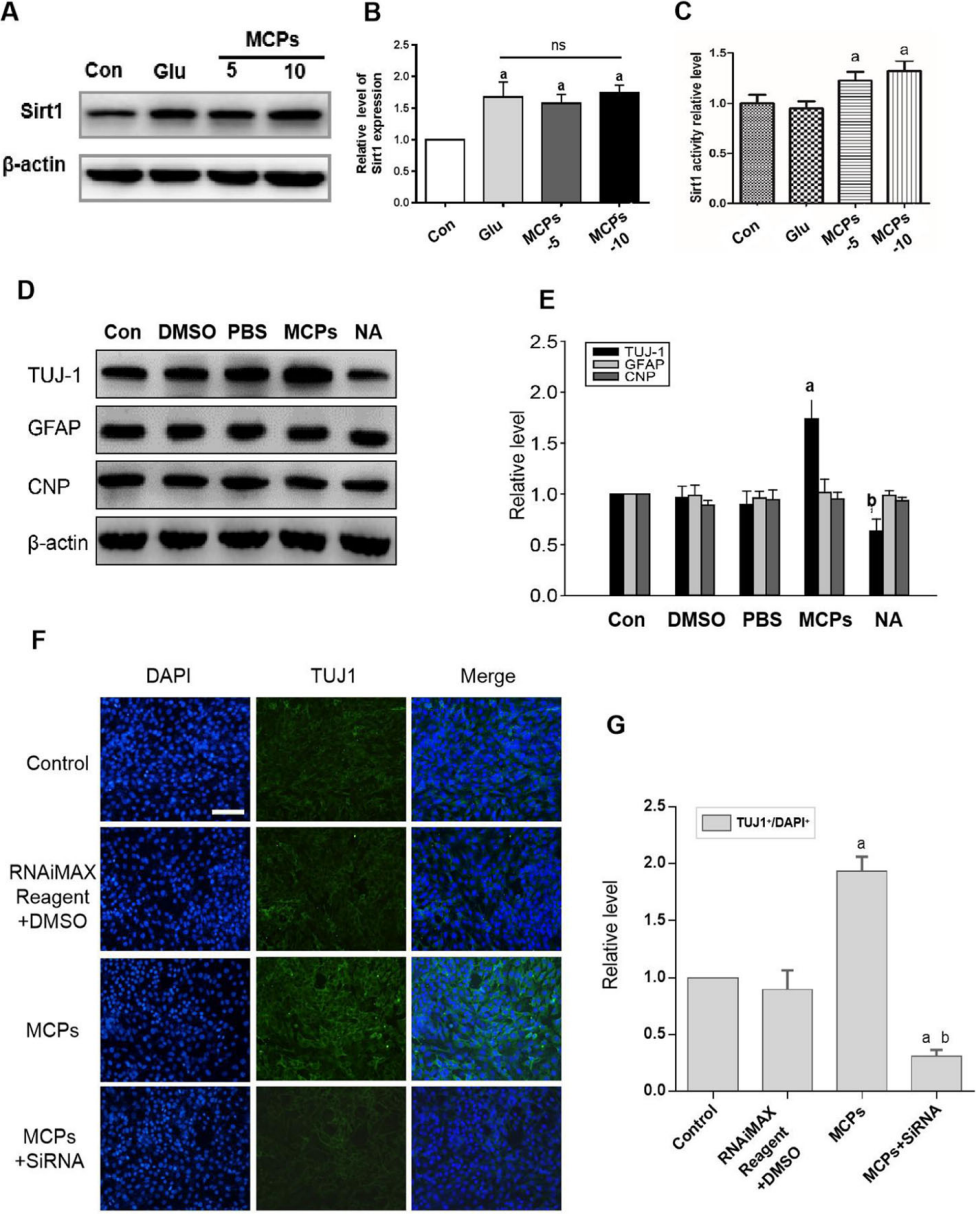
Activation of SIRT1 inducing by MCPs is responsible for the neuronal differentiation of C17.2-NSCs. **a** Western immunoblots of SIRT1 from indicated group. **b** Data were presented as the relative density of SIRT1 compared with that of β-actin from indicated group. **c** Activity of SIRT1 in C17.2-NSC differentiation by SIRT1 activity assay. **d** Western blots for TUJ1, GFAP, and CNP from indicated group. **e** Data were presented as the relative density of TUJ1, GFAP, and CNP compared with that of β-actin. **f** Immunofluorescence staining of TUJ1 (green) and DAPI (blue) in C17.2-NSCs from indicated group. Scale bar, 100 μm. **g** Quantitative analysis showing the percentage of TUJ1^+^ cells in indicated group. Data were given as mean ± SD according to ANOVA with *n* = 3 independent experiments in triplicate per group. ^a^*P* < 0.05 vs. Con, ^b^*P* < 0.05 vs. MCP group

For insight into further evaluating neuronal differentiation mechanisms of MCP treatment in C17.2-NSCs post glutamate stimulation, we measured SIRT1 protein expression by Western blot. As shown in Fig. 4a and b, the expression of SIRT1 in Glu group was obviously higher compared to that of Con group. Similarly, in MCP group, it was significantly increased compared with Con group. But MCP administration did not remarkably affect SIRT1 protein expression when compared to that in Glu group. Next, we detected the activity of SIRT1 by SIRT1 activity assay. The result (Fig. 4c) showed that the activity of SIRT1 in both concentration of MCP groups were upregulated compared with Glu group, indicating that MCP treatment enhanced activity of SIRT1.

In order to further study the role of SIRT1 in MCPs induced neuronal differentiation, the blockage of SIRT1 activity by nicotinamide (SIRT1 inhibitors) were used to investigated NSC differentiation. Western blot result showed that MCPs enhanced TUJ1 expression; in contrast, the nicotinamide (NA) abolished the promotion effect of MCPs on neuronal differentiation (Fig. 4d, e). Additionally, we used SIRT1-siRNA (represented as MCP + siRNA group) to suppress the expression of SIRT1 in the differentiation process and then detected the immunofluorescence staining of TUJ1 in C17.2-NSCs after glutamate treatment (Fig. 4f). As shown in Fig. 4f and g, MCPs strongly upregulated the number of TUJ1-positive cells, while this effect was significantly reversed by SIRT1-siRNA treatment. These data clearly indicate that MCP-mediated neuronal differentiation of NSC under ischemic/reperfusion injury (IRI) pathological conditions is related to the upregulating SIRT1 activity.

### MCP-induced deacetylation via SIRT1 promotes nuclear accumulation of β-catenin in NSCs

**Fig. 5:**
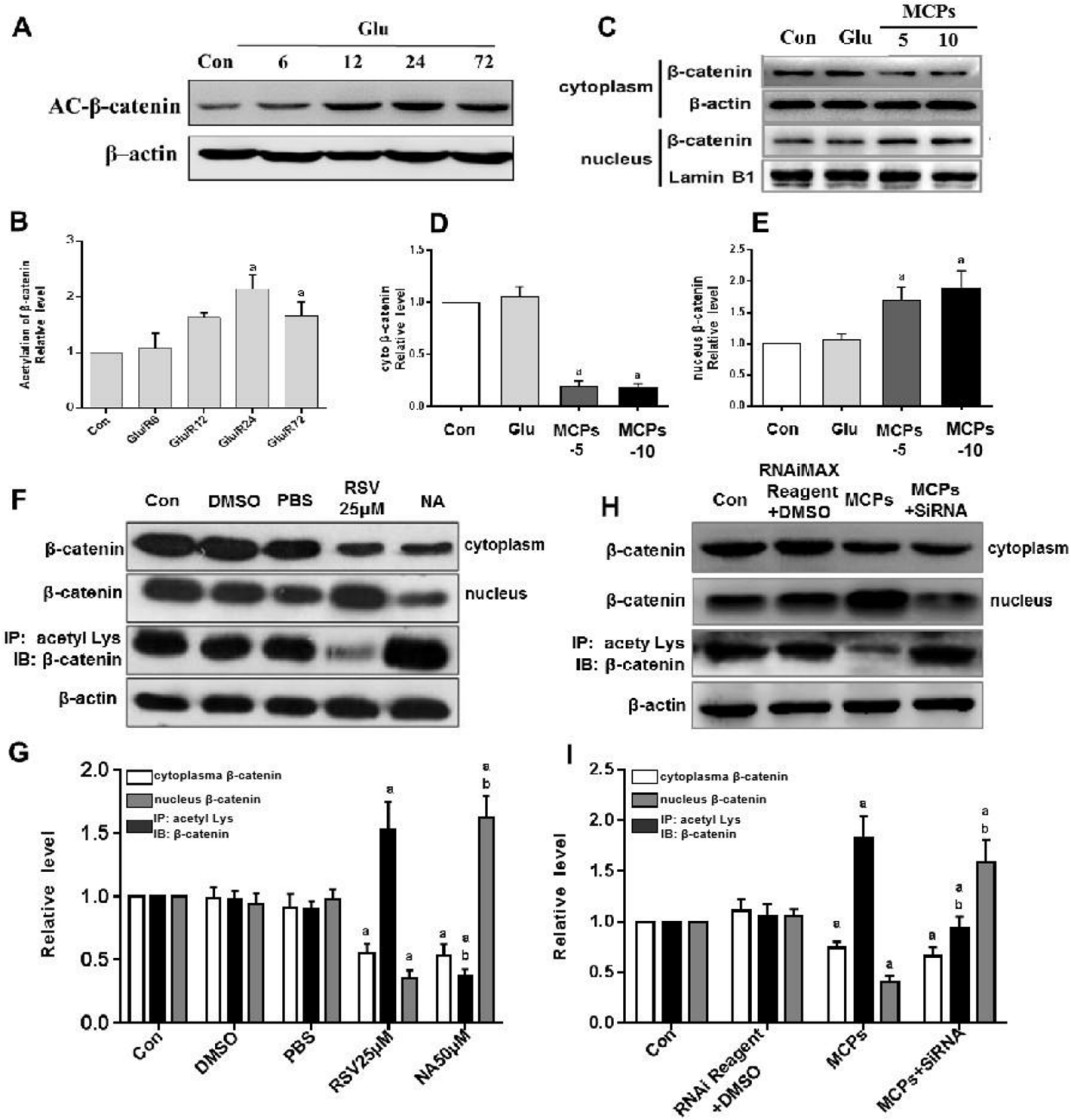
MCP-induced deacetylations via SIRT1 promote nuclear accumulation of β-catenin in C17.2-NSCs. **a** Western immunoblots of Ac-β-catenin after glutamic acid stimulation reperfusion of indicated time point. **b** Quantitative analysis of the expression of Ac-β-catenin compared with that of β-actin from indicated group. **c** Fractionated extracts analysis. Western immunoblots of β-catenin in nuclear and cytoplasmic from indicated group. **d**, **e** Statistical analysis for β-catenin nuclear accumulation. β-actin or LaminB1 was used for signal normalization. **f**, **h** Western immunoblots of the nuclear accumulation of β-catenin and Ac-β-catenin (IP, acety Lys; IB, β-catenin) from indicated group in glutamate-treated C17.2-NSCs. **g** Quantitative analysis of the nuclear accumulation of β-catenin and Ac-β-catenin treatment with RSV or NA (**f**). **i** Quantitative analysis of the nuclear accumulation of β-catenin and Ac-β-catenin treatment with MCPs or SIRT1-siRNA (**h**). Data were given as mean ± SD according to ANOVA with *n* = 3 independent experiments in triplicate per group. ^a^*P* < 0.05 vs. Con, ^b^*P* < 0.05 vs. RSV25μM or MCP group

The level of acetylation β-catenin was measured by Western blot analysis in C17.2-NSCs under ischemia-mimic condition. The results revealed that, as the time of reperfusion was prolonged, the level of acetylation β-catenin was significantly increased (Fig. 5a, b). Then, we tested the subcellular distribution of β-catenin in nuclear and cytoplasmic, respectively. The results showed that treatment with MCPs (5 or 10 μM) in C17.2-NSCs strongly resulted in a decrease in cytoplasm presence of β-catenin and an increase nuclear accumulation of β-catenin (Fig. 5c–e). Collectively, the above results implied that MCPs could affect distribution of β-catenin, and it may be closely related to the level of β-catenin acetylation via SIRT1. To confirm whether SIRT1 could regulate nuclear accumulation of β-catenin, resveratrol, an agonist of SIRT1, was used as the positive control for the evaluation of the effect of MCPs. As demonstrated in Fig. 5f and g, resveratrol significantly reduced the acetylation level of β-catenin and promoted its nuclear migration under the glutamate insults in C17.2-NSCs, while the nicotinamide, SIRT1 deacetylase inhibitor, increased the acetylation level of β-catenin and inhibited intracellular migration of β-catenin. To further validate whether the MCPs could play the same effect, we detected the acetylation levels of β-catenin in the presence of MCPs or SIRT1 siRNA and observed that MCPs remarkably downregulated the acetylation level of β-catenin and promoted its nuclear accumulation which were reversed by the SIRT1 siRNA (Fig. 5h, i). These results confirmed that effect of MCPs was mediated by the SIRT1/β-catenin axis.

## Discussion

In this study, our results revealed that MCPs could promote NSC neurogenesis after ischemic stroke. Our in vivo and vitro results identified previously unrecognized mechanisms that MCPs led to the activation of SIRT1 under glutamate-induced injury, which positively enhanced the nuclear accumulation of β-catenin and contributed to NSC differentiation into neuron in mimic IRI cell model. These findings indicate that MCPs may have the potential as a protective agent on late-stage recovery of ischemic stroke in clinical practice.

Recent years, polysaccharides have been widely used in medicine because of their low toxicity and obvious pharmacological effects [16]. As natural compounds found in daily foods, water extract of MCPs is well known for its anti-oxidation, anti-inflammation, anti-tumor, hypoglycemic, and anti-diabetic functions [13]. Several reports have shown that MCP treatment ameliorates oxidative stress injury in cardiovascular and cerebrovascular disease [38]. We have previously investigated that MCPs had a protective role on nerve injury after stroke by scavenging free radicals [11]. However, the mechanism underlying MCP-mediated neuroprotection in stroke and its pro-differentiation effect on injured brain tissues is still far from being understood. This is the first investigation detailing the neuronal differentiation action of MCPs in IRI.

Firstly, we observed that MCP can promote the differentiation of neural stem cells into neurons in SGZ region of MCAO model. We subsequently conducted in vitro study to explore the molecular mechanism of these important findings. To gain insight into the molecular mechanisms of the differentiation effect of MCPs on endogenous NSCs, an immortalized murine-derived neural stem cell line, C17.2-NSCs, was treated with different concentration of MCPs and then were detected the expression of NSC differentiation marker genes. We observed MCPs had no effects on the differentiation of neural stem cells under physiological conditions. This result suggests that MCPs (at different concentration ranges, 1.0, 3.0, 5.0, 10.0, 20.0 μg/ml) is safe for normal physiological NSCs and indicates that such concentration ranges may be physiologically acceptable for cultured cells in vitro. Next, in order to exclude the effect of C17.2 cells themselves differentiation on the experiment and to determine the time point of MCP administration, C17.2-NSCs were treated with glutamate and then were restored for different periods of time to mimic IRI. According to this experiment, we found that the differentiation features of C17.2-NSCs in IRI are consistent with previous reports. In glutamate-induced injury experiment, we aimed to determine the time points when there were the most robust damage effects (Fig. 3a, b). Therefore, we selected 3 days after glutamate treatment to evaluate intervention effects of MCPs. When MCPs was administered to C17.2-NSCs after glutamate-incubation, we observed that MCPs (2.0, 5.0, 10.0 μM) could obviously promote C17.2-NSC differentiation into neuron, in contrast, the astroglial differentiation of C17.2-NSCs were significantly inhibited. However, it seems that MCP concentration does not influence results. These data indicates that the range of effective MCP concentration is wide and has no concentration-dependent effects in our experiment system, and that these concentrations of MCPs do not reach the threshold is yet another possibility.

Astrocytes are the most abundant cells in the CNS and reactive astrogliosis accompany many pathological situations that affect the CNS, such as ischemic damage [39]. Compared with nonreactive astrocytes, reactive astrocytes show either deleterious effects on the progression of tissue damage or beneficial roles during recovery and repair. During recovery stage after IR, overactivated astrocytes can induce multiple damage effects and inhibit survival of newborn neurons [40]. The results of this study appear to suggest that the protective roles of MCPs are to enhance the neuronal differentiation of NSCs on one hand, and another possibility may be that MCPs could change astroglial differentiation which is more important in the recovery stage of CNS injuries. This effect of MCPs on inhibiting the over-activation of glial cells could potentially improve the microenvironment of NSCs survival or could promote neurite outgrowth and synaptic formation during brain repair stage. We have also investigated another primary neural stem cell derived from rat brain, E16-NSCs, and found that MCPs obviously enhance the neuronal differentiation, which strongly supported the results observed from immortalized C17.2-NSCs.

In latest years, therapeutic ability of NSCs has been matter of intense research, fueled by the hope of exploiting their regenerative potential for the treatment of different kinds of neurological impairments [41, 42]. Adult neurogenesis is the process whereby NSCs mature, migrate, and functionally integrate into the pre-existing neuronal network of an adult brain. Thus, the regenerative potential of endogenous neural stem cells in the adult brain has been proved as a likely source for regenerative tool to compensate for neuronal damage after ischemia stroke [43]. However, the molecular mechanisms underlying the regulation of neurogenesis in ischemic stroke remain to be elucidated. Our previous reports have demonstrated that MCPs mitigated ischemic stroke-induced damages by effectively eliminating oxygen free radicals and inhibiting JNK3 activation [11]. Our team has conducted research on the regulation of MCPs on neurogenesis after cerebral ischemia, and we will elaborate the proliferation effect of MCP on NSCs in another article. Our object of the present study is to focus on effect of MCPs on neuronal differentiation of NSCs under ischemia reperfusion injury and to understand its underlying mechanism.

In addition, to date, seven SIRTs in mammals (SIRT1–SIRT7) have been identified in different subcellular organelles and mediate different cellular functions depending on their typical substrate [24, 44, 45]. Interestingly, due to important effects on metabolism and physiological activities in CNS, SIRT1 has attracted great attention as medicinal targets particularly on neurodegenerative diseases [46]. Mountains of works have confirmed that SIRT1 regulates a wide variety of biological functions and cellular processes through the deacetylation of several histone/non-histone protein residues [47]. More importantly, cross talk between the gene regulation pathways of multiple transcription factors and SIRT1 determines the expansion and differentiation of stem cells [48]. In general, SIRT1 has drawn more attention because it is deemed to be one of the determining factors of the biology of stem cells. Although increasing evidence has demonstrate that SIRT1 plays a prominent role in the survival of differentiated neurons under various cellular stresses, some other studies have shown different findings. A review article of Cai et al. analyzed the opposing effects of SIRT1 on NSC lineage choice. They suggested a possible explanation that SIRT1 can be neuroprotective or neurotoxic depending on conditions, cellular stress, and cellular type. Another study also demonstrated that upregulation of SIRT1 repressed the proliferation of NPCs and directed their differentiation toward the astroglial lineage at the expense of the neuronal lineage under oxidative stress conditions in vitro and in vivo [49]. The conclusion emphasized the importance that SIRT1 function in NSC differentiation depends heavily on the redox state of the cells [24].

With respect to that, MCPs display free-radical scavenging properties; of course, it is entirely possible that MCPs change the redox state of the cells ultimately enhancing the activity of SIRT1. Therefore, in order to test whether the differentiation effect of MCPs was due to SIRT1 in more detail, we showed that MCP administration did not remarkably affect SIRT1 protein expression. But our further studies demonstrated that the activity of SIRT1 in MCP treatment group was upregulated compared with glutamate treatment group, indicating that MCP treatment enhanced activity of SIRT1 during the differentiation process. We also observed that when administrating with MCPs, NSC differentiation was promoted into neuron. In contrast, subjecting to NA (a known SIRT1 inhibitor) or knocking down SIRT1 with SIRT1 siRNA, NSC neuronal differentiation was significant downregulated. These results suggested that SIRT1 activation may contribute to the neuronal differentiation effects of MCPs under the ischemic-linked pathological conditions. One possible explanation for this finding is that the strong anti-oxidation of MCPs might change the intracellular redox state of NSCs after glutamate-induced injury, and SIRT1 might be more prone to play its deacetylase activity. This may explain our observation of MCPs promoting neuronal differentiation of NSCs in pathological models, accompanied by an increase in SIRT1 activity.

In our study as discussed above, SIRT1 is critical to MCP-induced NSC differentiation; however, what function might SIRT1 exert there? It is important to note that β-catenin is a deacetylation substrate of SIRT1, and acetylation/deacetylation modification of β-catenin participates in regulating its protein stability and transcriptional activity [50]. Recently, Leonard Guarente’s study showed that SIRT1 could deacetylates β-catenin and promotes its nuclear localization and activity in mesenchymal progenitor cells [51]. Intriguingly, numerous deacetylation substrates of SIRT1 have been identified, for example, one of them is β-catenin, the important transcription factor in Wnt signaling pathway [52, 53]. It is well documented that the protein stability and nuclear localization of β-catenin are required for Wnt pathway target genes, and usually some of those genes are closely related to neurogenesis. Several studies have validated that deacetylation of β-catenin by SIRT1 is correlated with β-catenin nuclear accumulation for its transcriptional activity [33, 50]. For example, a recent study done by Simic group reported that SIRT1-mediated deacetylation was required for nuclear accumulation of β-catenin in mesenchymal stem cells (MSCs) for their self-renewal and differentiation [51]. Therefore, we hypothesize that SIRT1/β-catenin axis participates in NSC differentiation via regulating Wnt signaling pathway in IRI. Therefore, in the final part of study, we observed a significant increased acetylation of β-catenin in glutamate-induced injury of the C17.2-NSCs. More importantly, cell fractionation studies showed that MCP treatment in C17.2-NSCs strongly resulted in a decrease in cytoplasm presence of β-catenin and an increase nuclear accumulation of β-catenin. Further experiments were carried out to verify whether the regulatory effect of MCP on the cellular localization of β-catenin was related to SIRT1. We found that, along with hyperacetylated, β-catenin was decreased in nucleus upon SIRT1 inhibition after stimulation with glutamate. In contrast, incubating with MCPs or RSV (SIRT1 agonist) rescued the nuclear localization defect and reversed the hyperacetylation of β-catenin in C17.2-NSCs. This part of data strongly suggests that MCPs could induce nuclear accumulation of β-catenin via SIRT1 in C17.2-NSCs. And this effect of MCPs is probably related to deacetylase activity of SIRT1. Although the detailed mechanisms underlying this interesting phenomenon remain to be defined, one plausible explanation is that MCPs are distinct SIRT1 activators which suggest an important potential therapy for ischemic/reperfusion injury.

However, some limitations of our study should be noted. Whether the effect of MCPs on neuronal differentiation is a direct or indirect effect is unknown. In general, due to the large molecular mass and complex composition of polysaccharides, it is difficult to permeate across the blood-brain barrier. Although impaired blood-brain barrier (BBB) under ischemic conditions facilitates MCPs permeate across BBB, affecting the neuronal activities directly, the composition of MCP is complex and diverse which makes it difficult to trace MCPs systematically. To us, this will be an interesting subject for a future study.

## Conclusion

**Fig. 6:**
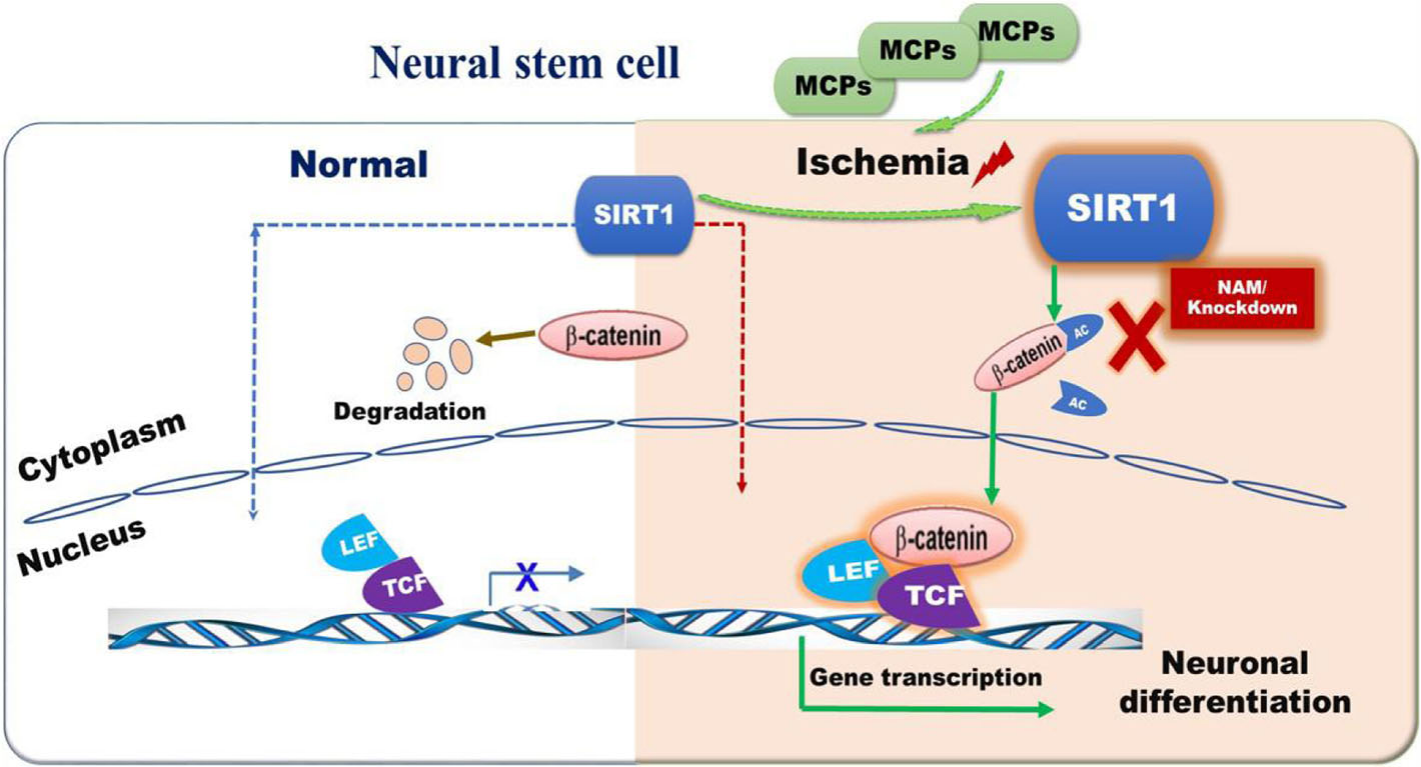
The mechanism graph of the regulatory network of MCPs/SIRT1/β-catenin in the neuronal differentiation of NSCs under glutamate-induced injury

In conclusion, our study demonstrates that MCPs have the potential to upregulate the activity of SIRT1 by changing intracellular redox state and subsequently motivate the deacetylation of β-catenin, thereby leading to the nuclear accumulation of β-catenin, consequently promoting the neuronal differentiation of NSCs in mimic IRI (Fig. 6). Thus, MCPs may have the potential as a novel nerval protective agent in clinical practice. Our study reveals a previously unknown effect of MCPs to promote neuronal differentiation via activating SIRT1/β-catenin pathway in IRI. This finding suggests that, in addition to its commonly known functions of anti-oxidation, MCPs also exhibit anti-inflammation, anti-tumor, and regulate neurogenesis effects. Our study suggests that water extract of MCPs as a potential therapeutic strategy in the treatment of ischemic stroke.

## Supplementary Information

**Additional file 1.**

## Data Availability

The datasets that support our conclusions of the current study are available from the corresponding author on reasonable request.

## Abbreviations

NSCs: Neural stem cells
IRI: Ischemia/reperfusion injury
MCPs: *Momordica charantia* polysaccharides
siRNA: Small interfering RNA
BBB: Blood-brain barrier
ROS: Reactive oxygen species
SIRT1: Silent information regulator 1
NPCs: Neural progenitor cells
