# Supplementary material for: Momordica charantia polysaccharides modulate the differentiation of neural stem cells via SIRT1/Β-catenin axis in cerebral ischemia/reperfusion
Source: Stem Cell Res Ther. 2020 Nov 16;11:485. doi: 10.1186/s13287-020-02000-2 (PMC7667795; doi:10.1186/s13287-020-02000-2)
Supplement: Supplementary file 1 — Additional file 1. [file 13287_2020_2000_MOESM1_ESM.docx]

**Supplementary figure S1.**


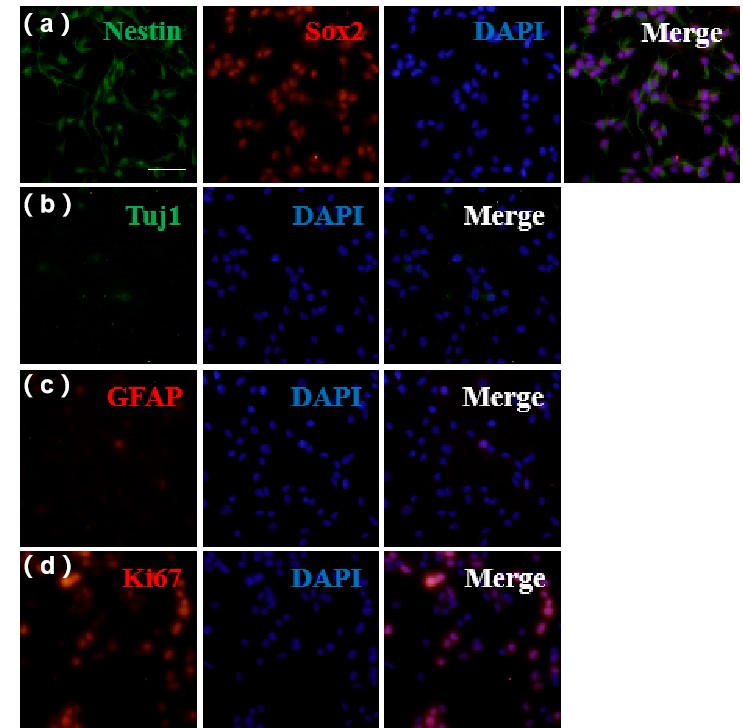


**S1. Characterization of E16-NSCs Phenotypic Properties**

Primary cortical neural stem cells (E16-NSC) grown at clonal density in medium generated epithelial-like clones positive for NSC markers. (a) Representative immunofluorescent images of Nestin (green) and SOX2(red) staining for NSC. (b) Representative immunofluorescent image of TUJ1 (green) staining for neuronal cells; (c) Representative images of GFAP (red) staining for astrocytes; (d) Representative immunofluorescent images of Kir67 (red) staining for the proliferation cells. DAPI was used to stain the cell nucleus (blue). Scale bar, 100 μm. n = 3 independent experiments in triplicate.

**Supplementary figure S2.**


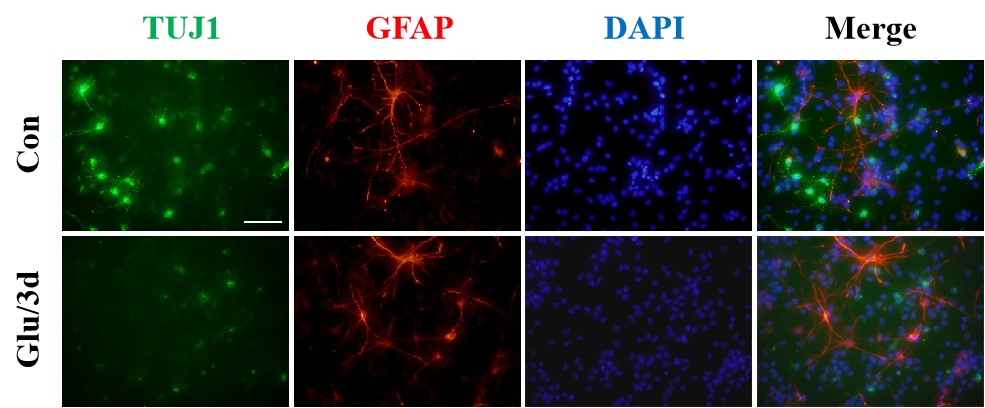


**S2. Characteristics of E16-NSCs cells after glutmate stimulation**

Immunostaining of Primary cortical neural stem cells (E16-NSC) after glutamate treatment. TUJ1 for neurons; GFAP for astrocytes. Nuclei were stained with DAPI (blue). Scale bars correspond to 100 μm. n = 3 independent experiments in triplicate.
